# Effect of self-management intervention on patients with chronic obstructive pulmonary diseases, Chitwan, Nepal

**DOI:** 10.1371/journal.pone.0296091

**Published:** 2024-01-02

**Authors:** Kalpana Sharma, Hem K. Subba, Sunita Poudyal, Shital Adhikari

**Affiliations:** 1 School of Nursing, Chitwan Medical College, Bharatpur, Nepal; 2 Department of Pulmonary and Critical Care Medicine, Chitwan Medical College, Bharatpur, Nepal; Gulu University, UGANDA

## Abstract

**Background:**

Self-management skills are important for patients with Chronic Obstructive Pulmonary Disease (COPD) who are responsible for their day to day care. Poor self-management behaviours have a significant influence on symptoms, functional impairments and quality of life. Evidence has shown that self-management interventions support patients to respond to changing symptoms and thereby make appropriate decisions regarding their self-management.

**Objective:**

This study aimed to find out the effect of self-management interventions in patients with COPD in terms of self-management practice, inhaler practice, COPD symptoms burden, functional ability, self-perceived dyspnoea and emotional symptoms.

**Methods:**

Quasi-experimental pre-test post-test design was carried out among patients with COPD attending respiratory units of Chitwan Medical College Teaching Hospital (CMC-TH), Nepal. Convenience sampling technique was used to select the 70 patients with COPD for the study. Baseline data was collected from the participants using (i) Semi-structured interview schedule for socio-demographic and clinical variables, (ii) COPD Self-Management Practice Questionnaire, (iii) Borg Dyspnoea Scale, and (iv) Six Minute Walking Distance (6MWD) Test (v) Pulmonary Function Test (PFT) and (vi) Observation Checklist. Self-management Intervention given was 2 ½ hour sessions per week for 6 weeks along with information booklets distribution. Participants were re-evaluated after 3 months of intervention using same tools. Data analysis was performed using IBMSPSS version 20.0 for window. Wilcoxon signed-rank test was performed to find the effectiveness of the self-management interventions on outcome parameters.

**Results:**

Self-management interventions (2 ½ hour session per week for 6 weeks) elicited a statistically significant change on self-management practice (z = -7.215, p<0.001), inhaler practice (DPI practice z = -6.731, p<0.001, MDI practice, z = -1.816, p = 0.005), functional ability (z = -4.243, p<0.001), self-perceived dyspnoea (z = -4.443, p<0.001), COPD symptom burden (z = -7.009, p<0.001) and emotional symptoms (depression, z = -6.856, p<0.001, anxiety, z = -6.675, p<0.001) of patients with COPD.

**Conclusions:**

Self-management intervention acts as powerful equipment to improve self-management practice, COPD symptoms burden, functional ability, self-perceived dyspnoea and emotional symptoms of patients with COPD. Hence, clinician and policy maker need to plan and intervene the rehabilitation program for the patients with COPD to enhance the effectiveness of therapy, self-management practice and general longevity.

## Introduction

Chronic Obstructive pulmonary Disease (COPD) is an important preventable and treatable public health challenge listed as one of the top three cause of death worldwide [1]. Around 90% deaths from COPD occurred in low- and middle-income countries [2]. In Nepal, the COPD prevalence ranges from 23%–43% [3–5] and is in increasing trend [6].

COPD remains as an irreversible disease which puts an immense burden on patients, healthcare workers and society as well. Self-management behaviour is crucial to delay the disease progression and avoid exacerbations and hospitalizations. Several studies has been reported that self-management intervention can improve the COPD patient’s lifestyle, reduce the costs of hospitalization, improve the patient’s depression and quality of life, reduce the emergency visits, improve knowledge and severity of disease, and increase self-efficacy of the patients [7–9]. However, poor self-management behaviours are believed to have a significant influence on symptoms, functional impairments and quality of life [10]. In addition, self-management strategies have also shown to increase medication adherence, increase physical performance and physical activity, increase use of breathing regulation techniques and energy-saving strategies during activities of daily living as well as to reduce the impact of COPD in daily life and breathlessness for individuals with COPD [11, 12]. Author also recommended health education for the patients to enhance their knowledge regarding disease process thereby help to improve self-management behaviour [13].

Evidence reported that COPD patients have low self-care management practice in Nepal and various factors such as inadequate infrastructure and resources, limited skills of primary level care providers and lack of educational materials for COPD are associated with it [14]. In addition, there is dearth of information regarding self-management intervention studies in Nepalese context. Hence, this study aimed to find out the effect of self-management intervention on patients with COPD attending Chitwan Medical College Teaching Hospital.

## Material and methods

### Study design

Quasi-experimental one group pre-test and post-test design was applied to assess the effect of self-management intervention in patients with COPD.

### Study setting

This study was carried out at the respiratory and critical care unit of Chitwan Medical College Teaching Hospital (CMC-TH) Bharatpur-10, Chitwan, This hospital is a tertiary care hospital with 650 beds, located in the central part of Nepal. Patients from different geographical areas attend for the treatment. The pulmonary and critical care department runs outpatient clinic 6 days a week. On an average 10–15 COPD patients per day attend in Out Patients Department (OPD) and total 100 patients with stage I to III COPD attended in OPD during 3months period.

### Study period

Total duration of study period was 2 years and data were collected from 1^st^ February 2022 to 31^st^ October 2022.

### Study population and eligibility criteria

Population of the study were those COPD patients who were clinically diagnosed to have COPD based on GOLD diagnostic criteria (FEV1/FVC ratio <0.7) and attending at CMC-TH during data collection period. This study included those patients who met the following criteria: (i) Patients who were diagnosed as COPD cases based on spirometry (FEV1/FVC ratio <0.7) and has history of illness for at least 3 months, and were in stable conditions (ii) age ≥18 years (iii) has GOLD stage III or less and (iv) patients who has undergone regular treatment for COPD for at least 3 months (iv) Patients who provided consent for the study. Those patients who had (i) problems in seeing, hearing and speaking (ii)severe co-morbidities in the heart, lungs, liver, kidneys and nervous system, and or any psychiatric disorders were excluded from the study.

#### Sample size determination and sampling techniques

A needed sample size of patients was calculated taking 86.1% prevalence (p = 0.86) [13], 95% confidence level (z = 1.96), 5% allowable errors (d = 0.05), and the total number of 100 COPD patients in the last 3 months (N = 100). For initial sample size (n_0_), following Cochran’s formula (1977) [15] was used i.e. n_0 = (Z)_^2^*p *q / d^2^ and calculated sample size was 186. After adjusting population using following formula, n = (N × n_0_) / {N + (n_0_ + 1)}, and 10% non-response rate, final sample size was 72.

Non-probability convenience sampling technique was used to select the desired sample. Those patients who came during data collection period and met the study criteria were taken as study sample.

### Operational definitions

#### Patients with COPD

It referred to those patients who were clinically diagnosed to have COPD for at least three months and attending at respiratory and critical care units of Chitwan Medical College Teaching Hospital (CMC-TH) for follow-up visits. The diagnostic criterion for COPD was used as per Global Initiatives for Chronic Obstructive Lung Diseases [16].

- presence of dyspnoea, chronic cough or sputum production, a history of recurrent lower respiratory tract infections and or history of exposure to risk factors for the diseases
- Clinically diagnosed COPD cases based on spirometry (FEV1/FVC ratio <0.70)

#### Self-management practice

It referred to activities and behaviours of patients with COPD to control and treat their conditions. It comprised of five distinct set of activities: (1) Symptom management (dyspnoea, cough sputum production) (ii) Daily life management (nutrition, medications, physical activities along with energy saving strategies, and use of breathing regulation technique) (iii) Information management (communication with clinician and accessing media) (iv) emotional management and (v) self-efficacy. It was assessed through 23 items self-management practice questionnaire

### Inhaler practice

It is referred to step wise performance of dry powder inhaler (DPI) or meter dose inhaler (MDI) by patients with COPD. It was assessed using observational checklist consisting total of 11 steps (2 critical steps and 9 general steps). Total score was calculated by summing score of all items and was further classified into two categories as:

Correct technique (Performing at least 90% of total steps including 2 critical steps)

Incorrect technique (Performing less than 90% of total steps)

#### Pulmonary function

It was measured through Spirometry parameters such as forced expiratory volume in 1 second (FEV_1_), and forced vital capacity (FVC) expressed both in absolute terms and as a percentage of the predicted value. Baseline value was obtained before intervention

#### Functional ability

It referred to aerobic capacity and endurance which was measured by using 6 minute walk test. The distance covered over a time of 6 minutes was used as the outcome by which to compare changes in performance capacity before and after intervention.

#### Self-perceived dyspnoea

It referred to the sensation of breathing difficulty experienced by the patient during their daily life activities. It was measured using a 5-point Modified Medical Research Council (mMRC) Dyspnoea Scale [17]. In addition, Modified Borg Dyspnoea Scale [18] was used to measure self-perceived dyspnoea during 6 minute walk test.

#### Emotional symptoms

It referred to anxiety and depression among patients with COPD which was measured using generalized anxiety disorder-7 and patients health questionnaire-9.

### Data collection tools and measurement

Three types of tools were used for the study: Interview schedule, Spirometry and Six Minute Walk Distance Test (6MWDT). *Interview Schedule* consisted of semi-structured questionnaire, Modified Medical Research Council (mMRC) Dyspnoea Scale, Borg Dyspnoea Scale, COPD Self-Management Practice Questionnaire, and COPD Assessment Test (CAT), and Patient Health Questionnaire9 (PHQ-9) and Generalized Anxiety Disorders (GAD-7).

*Semi-structured Interview Schedule* was developed for socio-demographic information and clinical information of the respondents. It included sex, age, education level, occupation, marital status, living status, place of residence, monthly household income, smoking status, medical insurance, disease duration, GOLD stage, hospital admission during the past 6 months and co morbidities.

*Modified Medical Research Council (mMRC) Dyspnoea Scale* [17] was used to stratify the severity of dyspnoea in patients with COPD. In this, patients with COPD were asked to rate their dyspnoea level associated with their daily activities. Different statements describing decreasing levels of physical activity that may precipitate shortness of breath are presented on a five-point scale (range 0–4), where 0-breathless on strenuous exercise, 1-only get shortness of breath when hurrying on level ground or up a slight hill, 2-I walk slower than people of same age on the level or have to stop for breath when walking at my own pace on the level, 3-I stop for breath after walking about 100 yards or after a few minutes on the level, I am too breathless to leave the house or I am breathless when dressing.

*Modified Borg Dyspnoea Scale (MBDS)* developed by Borg (1998) [18] was used to measure self-perceived dyspnoea as reported by patients during physical exercise i.e. 6MWD test. It consists of 0 to 10 rated numerical score that asks patients to rate the difficulty of their breathing. It starts at number 0 where there is no breathing difficulty at all and progresses through to number 10 where breathing difficulty is maximal. Patients were asked as “How much difficulty is your breathing causing you right now?”

*Self-Management Practice Questionnaire* was developed by adopting 18 items of *Self-Management Practice Questionnaire (*SMPQ) [14] of Yadav et al. and 5 additional items were added based on the review of literatures. This scale included 23 items categorize in 5 domains: symptom management (6 items), daily life management (8 items), emotion management (5 items), information management (3 items), and self-efficacy (1 item). The responses to each item were graded on 5-point scales where 1 = never, 2 = rarely, 3 = sometimes, 4 = often, and 5 = always. The SMPQ is applied in Nepalese context with good validity and reliability i.e. Cronbach alpha coefficient was above 0.7 indicating good internal consistency [14]. Pretesting of the instrument was done among 50 COPD patients attending medicine OPD of CMC-TH and reliability of the instrument was tested by calculating Cronbach’s alpha values which was 0.83

*COPD Assessment Test (CAT) scale developed by* Jones et al. (2009) [19] *was* used to assess the COPD symptom burden. It consisted of eight items (cough, phlegm, chest tightness, breathlessness, activities, confidence, sleep and energy) rated on a 0 to 5 scale. Total score was 0–40 with higher score indicating a higher symptom burden.

*Patient Health Questionnaire-9(PHQ-9) and* Generalized Anxiety Disorders-7 (GAD-7) were used for the screening of emotional symptoms among patients with COPD. GAD-7 was used for the assessment of anxiety [20] and PHQ-9 for depression [21]. These instruments assessed the symptoms experienced by patients during the 2 weeks before they took the survey. Each item of GAD-7 and PHQ-9 was rated 0 to 3 scores where 0-not at all, 1-several days, 2-more than half of the days, and 3-nearly every day, with higher scores indicating patients’ increased self-report of anxiety and depression severity.

*Spirometer (Model RMS Hellos 401)* was used to measure baseline pulmonary function of the respondents. COPD disease severity was classified into four categories according to GOLD criteria as:

Mild (I): Post-bronchodilator FEV_1_/FVC <0.7, FEV_1_≥80% predicted

Moderate (II): Post-bronchodilator FEV_1_/FVC <0.7, FEV_1_**≈**50%–80% predicted

Severe (III): Post-bronchodilator FEV_1_/FVC <0.7, FEV_1_**≈**30%–50%predicted

Very severe (IV): Post-bronchodilator FEV_1_/FVC <0.7, FEV_1_<30%predicted

*Six Minutes Walking Distance Test (6MWDT)* was used to assess the functional capacity of the patients with COPD. American Thoracic Society (ATS) [22] guideline was followed for the 6MWD test. Walking track was same layout for all tests for patients. The tract was a point to point (stop, turn around, go) track. The track was flat, with minimal obstacles. The minimum length of the walking tract was 25 meters.

*Observational check-list* was used to assess the inhaler use among patients with COPD. Observational checklist consisted of total 11 steps (2 critical steps and 9 general steps). Total score was 0–11. Each statement was rated to 0 to 1 score, where 0- incorrectly performed and 1-correctly performed.

### Interventions description

Intervention training package was designed for the people living with COPD to provide information/education and skills regarding self-management practice of COPD. It was developed based on the extensive literature review and consulting with respiratory and critical care specialists. It included four components: (i) education components on disease and self-care management (ii) training on inhaler technique, (iii) 6 minutes’ walking distance (iv) deep breathing and coughing exercises.

Participants were classified into 6 groups (12 in each group). Each group received 6 training sessions from the experts in the forms of lectures, video clip display, group discussion, demonstration and individual counselling. Each session was conducted for at least two and half hour/day and session was provided in weekly basis for 6 weeks. Further, telephone follow-up was carried out for the patients in monthly basis to reinforce to follow proper instructions and training. The post-test (functional capacity, self-management practice, inhaler practice, self-perceived dyspnoea)was done by researchers after three months of interventions.

Day for intervention was set for Sunday to Friday from 12 Noon to 2.30 PM. Educational and training sessions were planned and conducted in room located in the OPD of CMC-TH. Each intervention sessions were led by research team members by themselves. Before intervention participants were introduced to each other and with research team members. They were also explained about the process of intervention, their role during the intervention and after the intervention. Further, they were assured that if any complications arise they can contact to research team member through the telephone from 10 am to 4 pm for advice and referral to hospital if needed. Face-to-face conversations were taken among the experts and participants during intervention. Each session was started with the participants’ everyday life experiences. Themes such as problem solving, goal setting, symptoms analysis, social challenges, physical activity, nutrition, medication, smoking cessation, exacerbations, and psychological issues were mentioned. A small booklet with information and advice about symptoms management, nutrition, physical activity, and health care resources were given to the participants after the completion of self-management interventions.

### Data collection

Data were collected by researchers themselves through interview, observation and bio-physiological measurement from 1^st^ February 2022AD to 31^st^ October 2022 AD. First of all, participants were evaluated through spirometer (model Hellos 401) and disease severity was classified according to GOLD criteria as mild, moderate, severe and very severe. After identification of possible sample and obtaining written informed consent, socio-demographic, clinical and COPD knowledge related information were collected from the patients. After proper history, initial assessment of COPD self-management practice, and 6 minute walking distance tests (6MWDT) were done. Participants’ contact numbers were collected. Participants were explained for the intervention program, time and date. Self-management interventions program was implemented as schedule. Participants’ 6MWDT along with self-management practice were re-assessed after 3 months of intervention.

### Data quality control

The questionnaire were translated into Nepali language and back translated to English language by language expert for the consistency. The questionnaire was pre-tested among 10 COPD patients attending Medicine OPD of CMC-TH but they were excluded for the final study. Nepali version tools were used for the data collection. Anthropometric and spirometry measurement were taken using standard protocol. Each anthropometric parameter was taken twice and the measurement was repeated if the difference existed. Two hours briefing session was arranged on the data collection process for the data collectors and spirometry measurement technician. Intervention was given by the researchers using similar booklets, and guideline procedure.

### Data management and statistical analysis

All the collected data were analysed using statistical software package SPSS version 23.0 for window. Descriptive statistics was performed to describe the population with regard to socio-demographics, clinical and baseline characteristics. Continuous data were presented as mean, standard deviation or median and interquartile range based on the nature of the data. Number and percentage were used for the presentation of categorical data. Wilcoxon signed-rank test was applied to compare two related samples, outcome measures in both the pre intervention phase and the post intervention phase. Level of statistical significant was set at p<0.05.

### Ethical considerations

Ethical clearance was taken from Institutional Review Committee of Nepal Health Research Council (Ref. No.1908). Data collection permission was obtained from Chitwan Medical College-Institutional Review Committee (Ref. No: CMC-IRC/078/079-055). Written Informed consent was taken from each respondent prior to data collection. There was no any type of physical and psychological harm to the participants during research. Dignity was maintained by giving option to discontinue from the research study at any time without any penalty. Privacy was maintained during data collection by interviewing them in separate room. Confidentiality was maintained by not disclosing the information to others and using code number instead of name of participants. Spirometry test was done for the respondents with the free of cost and results were provided to them. In addition, travel costs were provided for the participants during their intervention period.

## Results

Out of 72 participants involved in the pre-test and intervention, two participants did not involve in post-test so sampling mortality was 2.78%. Out of 70 participants, nearly half (48.2%) were belonged to the 65–74 years age group. The overall mean age (±SD) was 69.47 (±7.67) years. Majorities were females (61.4%), illiterate (52.9%), involved in agriculture (55.7%), and had monthly income just enough to support their family (68.6%). Most of the participants had taken health insurance facility (80.0%) and 52.9% quitted their job due to illness (Table 1).

**Table 1:** Socio- demographic characteristics of the participants. n = 70.

Variable	Number	Percentage
**Age group in years**		
50–64	15	21.4
65–74	34	48.6
75 and above	21	30.0
Mean age (SD): 69.47 (±7.67) year	Min:50 year	Max-85 year
**Sex**		
Male	27	38.6
Female	43	61.4
**Educational status**		
Illiterate	37	52.9
literate	33	47.1
**Occupation**		
Agriculture	39	55.7
Household work	16	22.9
Service	6	8.6
Business	5	7.1
Others	4	5.7
**Monthly family income**		
Not enough	12	17.1
Just enough	48	68.6
Surplus	10	14.3
**Health insurance facility**		
No	14	20.0
Yes	56	80.0
**Quitting of job due to illness**		
Yes	37	52.9
No	33	47.1

More than half of participants (52.9%) had COPD for more than 5 years with median duration of COPD (IQR) was 6 (3–8.25) year. Mean of FEV1/FVC ratio was 69.68 where minimum was 38.65 and maximum 69.0. Most of the participants (85.7%) had moderate stage of COPD. Majorities of the participants (88.6%) were smokers before illness whereas 17.2% were still smoking cigarette even after diagnosis of COPD, more than half (57.1%) had other chronic co-morbid conditions (57.1%), and Just half (50.0%) of the respondents had dyspnoea grade I and most of participants (85.7%) were DPI users (Table 2).

**Table 2:** Clinical characteristics of the participants. n = 70.

Variable	Number	Percentage
**Duration of COPD**		
Less than 1 year	9	12.9
1–5 year	24	34.3
Above 5 year	37	52.9
Median duration of COPD (IQR): 6 (3–8.25) year Min: 0.4 year Max: 35 year		
Mean % of FEV1/FVC (±SD): 69.68 (±8.15) Min: 38.65 Max: 69.0		
**GOLD stage**		
Moderate (50% ≤FEV1 <80%)	60	85.7
Severe (30% ≤ FEV1 <50% predicted)	10	14.3
**Smoking status before illness**		
Non smoker	8	11.4
Smoker	62	88.6
**Smoking status after illness**		
Non-smoker	8	11.4
Ex-smoker	50	71.4
Current Smoker	12	17.2
**Presence of co-morbidities**		
Yes	40	57.1
No	30	42.9
**Type of conditions (n = 40)**		
HTN	21	52.5
Arthritis	7	17.5
DM	9	22.5
Heart problem	5	12.5
others	3	7.5
**Degree of breathlessness through mMRC scale**		
One	35	50.0
Two	15	21.4
Three	20	28.6
**BMI**		
Undernourished	9	12.9
Normal	41	58.6
Overweight	16	22.9
Obese	4	5.7
**Type of inhaler use**		
Meter dose inhaler	10	14.3
Dry powder inhaler	60	85.7

Very few (4.3%) participants used inhaler correctly whereas almost all (95.7%) used inhaler incorrectly. In addition, more than one third of the participants suffered from moderate to severe anxiety (34.3%) and depression (42.8%). Likewise half experienced severe to very severe COPD symptom burden (50.0%) (Table 3).

**Table 3:** Baseline outcome parameters of participants. n = 70.

Baseline Outcome Parameters	No.(%)/Median(IQR)
**Inhaler practice**	
Inadequate	67 (95.7)
Adequate (>90% with 2 critical steps)	3 (4.3)
**Depression status**	
None (<5)	16 (22.9)
Mild (5 to <10)	24 (34,3)
Moderate (10 to <15)	17 (24.3)
Moderately severe (>15 to 20)	12 (17.1)
Severe (≥20)	1 (1.4)
**Anxiety status**	
None (<5)	23 (32.9)
Mild (5 to 9)	23 (32.9)
Moderate (10 to 14)	11 (15.7)
Severe (≥15)	13 (18.6)
**COPD symptoms burden (CAT questionnaire)**	
Mild (<11)	11(15.7)
Moderate (11–20)	24(34.3)
Severe (21–30)	29(41.4)
Very severe (31–40)	6(8.6)
**SMP**	61.0(52.7–68.2)
**Functional ability (6MWD in meter)**	200 (150–250)
**Perceived dyspnoea** through Borg Dyspnoea Scale	2.0 (0.5, 4.0)

A Wilcox signed ranked test showed that once weekly 2 and ½ hours sessions for 6 weeks self-management intervention elicit a statistically significant change on all the domains of self-management practice domains i.e. symptom management (z = -7.198, p<0.001), emotional management (z = - 6.998, p<0.001), information management (z = - 6.066, p<0.001), self-efficacy (-4.412, p<0.001), and overall self-management practice (z = -7.215, p<0.001) of respondents. Indeed, self-management practice was 61 and 82 respectively in pre-test and post intervention (Table 4).

**Table 4:** Difference on self-management practice before and after management. n = 70.

Variable	Pre Intervention Median (IQR)	Post Intervention Median (IQR)	p value	W (test value)	Difference in Mean Rank
Symptom management	34.0 (29.7, 39.0)	49 (46.7, 51.2)	<0.001	-7.198	33.99
Emotional management	11.0 (10.0, 14.0)	16 (15.0, 18.0)	<0.001	-6.998	29.96
Information management	10 (9,11)	12 (12,13)	<0.001	-6.066	13.99
Self-efficacy	4 (4,5)	5 (4,5)	<0.001	-4.412	1.7
Overall self-care management	61.0 (52.7,68.2)	82.0 (79,86)	<0.001	-7.215	34.5

A Wilcoxon signed-rank test showed that self-management intervention (2 ½ hour session per week for 6 weeks elicits a statistically significant change on inhaler practice (DPI practice z = -6.731, p = <0.001, MDI practice, z = -1.816, p = 0.005), emotional symptoms (depression, z = -6.856, p = <0.001, anxiety, z = -6.675, p = <0.001), functional ability (z = -4.243, p = <0.001), perceived dyspnoea (z = -4.443, p = <0.001), COPD symptom burden (z = -7.009, p = <0.001) and self-management practice (z = -7.215, p = <0.001) among participants (Table 5).

**Table 5:** Outcome parameters before and after self-management intervention.

Variable	Pre Self-management Intervention Median (IQR)	Post Self-management Intervention Median (IQR)	p value	W (test value)	Difference in Mean Rank
**Inhaler practice**					
MDI practice	6 (5, 7.5)	11(11,12)	0.005	-2.812	5.5
DPI practice	8 (7,10)	13 (13,13.7)	<0.001	-6.731	27.97
**Emotional symptoms**					
Depression (PHQ9)	8.0 (5,13)	2.0 (0,4)	<0.001	-6.856	11.24
Anxiety (GAD7)	6.0(3,7)	2.5 (0,4)	<0.001	-6.675	26.29
**Functional ability** through 6MWD in Meter	200 (150,250)	225(175,300)	<0.001	-4.243	0.73
**Perceived dyspnoea** through Borg Dyspnoea Scale	2.0 (0.5, 4.0)	0.5 (0,2)	<0.001	-4.443	9.36
**COPD symptoms burden** through CAT	20.5(13.5,27.2)	10.5 (4,16.2)	<0.001	-7.009	29.61
**Self-management practice**	61.0 (52.7,68.2)	82.0 (79,86)	<0.001	-7.215	34.5

## Discussion

Self-management is an important component for the effectiveness of the therapy of patients with COPD. Our study found that self-management interventions improves the self-management practice, inhaler practice, and functional ability of the patients as well as it is helpful to reduce emotional symptoms, COPD symptom burden, and self-perceive dyspnoea of COPD patients.

Our study revealed low self-management practice (61 out of 115) among patients and it is also consistence with the findings of the study done in Nepal which reported that SMPs among the sample of Nepalese with COPD were low [14]. Further, author highlighted the need for the implementation of a self-management intervention program involving patients’ activation and health literacy focused activities for COPD [14].

Correct inhalation technique is an important skill for the effectiveness of therapy for the COPD patients [1, 23]. However, only 4.3% of COPD patients of this study used the inhaler correctly whereas almost all (95.7%) used inhaler incorrectly. This finding is almost consistent to the finding of studies done by Poudel et al. [24] and Shrestha et al. [25] which showed 5.7% and 11.4% respectively of correct inhaler users. Similar findings have also been reported in other studies conducted in Nepal [26–28]. Incorrect use of inhaler might be due to patients’ lack of curiosity or learning attitude, and their negligence or inability to read the instruction pamphlet given with the drug package. In addition, inadequate instructions provided by the health providers might be other reasons for the incorrect inhaler use. Furthermore, evidence suggested that repetition, demonstration, and simplification form the foundation of effective inhaler training [29].

COPD patients are more prone to develop emotional problems compared to general population [30]. Consistent to this, 34.3% of patients with COPD had severe anxiety and 42.8% had moderate to severe depression. However our findings is slightly higher than the study done in India in which 28.4% of COPD patients had psychiatric co morbidity 9.5% was anxiety, and 8.1% was depression [30]. Likewise, Sharma et al. reported 10.6% anxiety and 13.8% depression among COPD patients in Nepalese context [31]. The higher frequency of anxiety and depression in our study might be due to use of different diagnostic tools and cut-off points.

This study showed the significant change on all the domains of self-management practice after self-management intervention program especially on symptom management emotional management information management, self-efficacy and overall self-management practice. Further, significant improvement was found on functional ability and inhaler practice whereas significant reduction on emotional symptoms, self-perceived dyspnoea and COPD symptom burden of patients with COPD after the self-management interventions.

In line with our findings a randomized control trial found that health mentoring delivered by community health nurse increased the self-management capacity of health mentor group (PIH overall 0.15, 95% CI 0.03 to 0.29; knowledge domain 0.25, 95% CI 0.00 to 0.50) whereas anxiety decreased in both groups (HADS A 0.35; 95% CI −0.65 to −0.04) and coping capacity improved (PIH coping 0.15; 95% CI 0.04 to 0.26) [32]. Likewise, another study proved that self-management education program improved the self-efficacy of COPD patients in the Chinese population of Macao as well as provide feeling of able to better control their disease and improve psychosocial well-being [33]. Further, Wang, Tan, Xiao and Deng also indicated the significant reductions in emotional distress in the SME group [34].

These findings are supported by a systematic review which showed the improvement in self-efficacy, anxiety and depression, and quality of life over the period of COPD self-management interventions [14]. Similarly, another randomized controlled trial in Norway showed the significant positive changes on constructive attitudes, approaches and skill and technique acquisition as well as some self-management related domains after the self-management support intervention i.e. better living with COPD [35]. Likewise, Hosseinzadeh and Shnaigat showed the positive outcome of COPD self-management trials on COPD knowledge and self-management behaviors such as adherence to medication, physical activities and smoking cessation in some cases [36]. Similarly study done in Australia found moderate improvement in physical activity level of COPD patients and their knowledge after health literacy interventions [37].

However, other studies revealed inconclusive findings on the some component of self-management outcome such as smoking cessation, medication adherence, dyspnoea, mental health, hospital admissions and health related quality of life [34, 37]. Likewise, one systematic review reported that self-management interventions delivered in the community to patients from primary care is not effective [38]. Furthermore, authors recommended identifying effective self-management interventions suitable for primary care populations, particularly those with milder disease [38]. This difference in findings might be due to different measuring tools, populations and intervention protocol on self-management intervention.

In our study, self-management intervention including one to one demonstration and re-demonstration of inhaler technique was effective to improve the inhaler practice of patients with COPD. This is consistent with the other study in which repeated face-to-face inhaler education by an advanced practice nurse was effective to improve the inhaler satisfaction, technique, and adherence [39]. This indicates that regular demonstration and re-demonstration of the inhaler techniques helps to improve inhalation skills of the COPD patients.

This study revealed that psychological problems such as anxiety and depression were reduced after receiving self-management intervention. This is supported by the studies which indicated significant reduction in emotional problems with the feeling of able to better control their disease after self-management education program [33, 34]. However, meta-analyses done by Jolly et al. showed contrast findings as there was no difference in anxiety and depression in patients with COPD from primary care in final follow-up after the supported self-management interventions [38]. This discrepancy in findings might be due to different self-management intervention package and measurement tools used in the studies.

To the best of our knowledge, this is the first study in the Nepalese context where self-management intervention package included education, training, demonstration, re-demonstration, leaflets distribution and follow-up for the patients with COPD. It adds to the dearth of information regarding effectiveness of the self-management interventions in patients with COPD. Spirometry evidence of air flow limitations was assessed for all patients for the enrolment into study so all the patients included in the study were clinically diagnosed and confirmed COPD patients. In spite of this, it has certain limitations: (i) This is a quasi-experimental one group pre-test post-test design which could not conclude the true cause and effect relationship (ii) This study was conducted in a single setting among OPD attended patients with COPD so the results may not be generalized to patients with very severe disease i.e. GOLD stage IV (iii). Certain confounding factors such as participants’ habit of physical activity could influence the follow-up results.

## Conclusions

This study is concluded that self-management intervention acts as powerful equipment to improve self-management practice, inhaler practice, and functional ability whereas reduce the COPD symptoms burden, self-perceived dyspnoea and emotional symptoms of patients with COPD. Hence, clinician and policy maker need to plan and implement the self-management intervention program on regular basis for the patients with COPD to enhance their self-management practice, effectiveness of therapy and general longevity.

## Supporting information

S1 File The questionnaire used for the data collection. (PDF)
