# Supplementary material for: Effect of self-management intervention on patients with chronic obstructive pulmonary diseases, Chitwan, Nepal
Source: PLoS One. 2024 Jan 2;19(1):e0296091. doi: 10.1371/journal.pone.0296091 (PMC10760700; doi:10.1371/journal.pone.0296091)
Supplement: S1 File — (PDF) [file pone.0296091.s001.pdf]

Interview Schedule on Effectiveness of Self-Management Intervention among Patients with COPD

Direction for Interviewer: This interview schedule has three major parts. Part I is related to socio-demographic information, disease related variables and health promotion activities of respondents. Questions in part I are in dichotomous form which need to be answered in **Yes or No** response. Part II is related to quality of life of respondents which includes 55 items in 5 point rating scale. There is only one appropriate response which needs to be ticked (√) in the provided box. Part III consists of open-ended questions which need to be described in the provided space.

Code Number: ………………………..

Place of Interview: CMC (Medical OPD)

Date of Interview:………………………..

Part 1

Socio-demographic Information

1. Age ……………….. (in years)
2. Sex: ☐ Male ☐ Female
3. Residence: Municipality/Sub metropolitan city/VDC
4. Ethnicity…………………………………………………
5. Religion………………………………………………..
6. Marital Status: Married/Unmarried/Widow/Widower
7. Type of Family: ☐ Nuclear ☐ Joint
8. Currently living wit: Single/Family/other………..
9. Educational Level ☐ Illiterate ☐ Literate

   If Literate,

   ☐ Just read and write

   ☐ Basic

   ☐ Secondary

   ☐ Bachelor and above

10. Occupation	Before Illness	After Illness	Remarks
☐ Farming	……………………	…………………..	………..
☐ Home Maker	……………………	……………………	…………
☐ Service	……………………	……………………	…………
☐ Business	……………………	……………………	………….
☐ Labour/daily wages	……………………	…………………….	………….
☐ Others……………..	……………………	……………………..	………….

10.1 Do you need to leave your job due to breathing problem? ☐ Yes ☐ No

11. 1. Economic Status

☐ Not enough to eat for one year
☐ Just enough to eat for one year
☐ Surplus for future

11.2 Income per months in NPR:

12. Duration of COPD (years since diagnosis)………………………….

Risk Factors of COPD

13. Smoking Status	Before Illness	After Illness	Remarks
a. Non Smoker	…………………..	…………………..	………….
b. Smoker	…………………..	…………………..	…………..
c. Ex-smoker	…………………..	…………………..	…………..

13.1 If smoker,

a. Age at which smoking was initiated…………………………

b. Total years of smoking………………………………………

c. Number of cigarettes used per day………………………….

d. Type of cigarette used: ☐ Cigarette ☐ Bidi ☐ Hookah ☐ Others……

14. Living with smoker (passive smoking)

a. Before diagnosis ☐ Yes ☐ No

b. After diagnosis ☐ Yes ☐ No

15. Exposure to dust, noxious gases or smokes in work place

a. Before diagnosis ☐ Yes ☐ No
b. After diagnosis ☐ Yes ☐ No

16. Types of fuel used for cooking at home: Wood or firewood/ Kerosene stove/ LP Gas/ Electricity/Others (specify)…….

17. Do you cook food at home? ☐ Yes ☐ No

Other Associated Health Problems (Co-morbid Conditions)

18. In addition to COPD, do you have any other health problems? ☐ Yes ☐ No

18.1 If yes, which of the following conditions do you have?

	Diabetes	☐ **Yes**	☐ **No**
	High blood pressure	☐ **Yes**	☐ **No**
	High cholesterol level	☐ **Yes**	☐ **No**
	Heart disease	☐ Yes	☐ No
	Asthma	☐ **Yes**	☐ **No**
	Renal Problem	☐ **Yes**	☐ **No**
	Arthritis	☐ Yes	☐ No
	Allergic Rhinitis	☐ Yes	☐ No
	Others (if specify)	…………	…………

Information obtained from Medical Record

19. Anthropometric Measurements

a. Weight (in kg).......................... b. Height (in cm)..........................

c. Body Mass Index (weight in kg/ height in m^2) = ……

d. Blood Pressure (mm of Hg)……………………………

20. Stage of COPD**:** Mild/moderate/severe/very severe

21. Severity of COPD (in case of spirometry)

a. FEV_1 /FVC (Liters)……………

b. Predicted FEV_1 (Liters)…………

22. Modified MRC Dyspnea Grade

**S. N.**	**Degree of breathlessness related to activity**	**Grade**
1	Only get breathless with strenuous exercise	0
2	Get short of breath when hurrying on a level or when walking up a slight hill	1
3	Walks slower than people of same age on the level, because of breathlessness, or have to stop for breath when walking on my own peace on the level	2
4	Stops for breath after walking 100 meters or after a few minutes on the level	3
5	Too breathless to leave the house, or breathless when dressing/undressing	4

Therapeutic Management

23. Medications used for breathing problems ☐ Yes ☐ No

If yes,

S.N	Lists of Medicine	Dose	Route	Duration (months or year)	Remarks
1					
2					
3					
4					
5					
6					

24. Does the current medication help you? ☐ Yes ☐ No

25. Do you need supplemental oxygen at home? ☐ Yes ☐ No

25.1 If yes,

a. Total duration of oxygen use at home (months or years)………………….

b. Frequency of oxygen use at home in last 3 months: Never/Occasionally/Sometimes/Most of time/Always

c. Total hours of oxygen use in 24 hours……………………………………..

26. Who is taking care of you during illness?..

Part 2: **COPD Assessment Test™ (CAT)**

For each item below, place a mark (X) in the box that best describes you currently. Be sure to only select one response for each question.

I never cough	0 1 2 3 4 5	I cough all the time	☐
I have no phlegm (mucus) in my chest at all	0 1 2 3 4 5	My chest is completely full of phlegm (mucus)	☐
My chest does not feel tight at all	0 1 2 3 4 5	My chest feels very tight	☐

When I walk up a hill or one flight of stairs I am not breathless	0 1 2 3 4 5	When I walk up a hill or one flight of stairs I am very breathless	☐
I am not limited doing any activities at home	0 1 2 3 4 5	I am very limited doing activities at home	☐
I am confident leaving my home despite my lung condition	0 1 2 3 4 5	I am not at all confident leaving my home because of my lung condition	☐
I sleep soundly	0 1 2 3 4 5	I don't sleep soundly because of my lung condition	☐
I have lots of energy	0 1 2 3 4 5	I have no energy at all	☐

Part 3

Six Minute Walk Test: **Borg Dyspnea Scale**

Scoring	Obtained scoring	Level of exertion
0		No Exertion
0.5		Very, very slight
1		Very slight
2		Slight
3		Moderate
4		Somewhat severe
5		Severe
6		
7		Very severe
8		
9		Very, very severe
10		Maximal

Part 4

Observation Checklist

Observation check-list on inhalation of Meter Dose Inhaler (MDI) Steps

	Steps	Performed	Not Performed
1	Remove the cap of inhaler and check the mouth piece thoroughly to see that it is clean	( )	( )
2	Shake the inhaler vigorously for about 10 seconds	( )	( )
3	Hold the inhaler upright with your index finger on the top	( )	( )
4	Sit up straight or stand and breathe out gently through mouth away from the inhaler immediately	( )	( )
5	Place the mouth piece in your teeth (do not bite) and grips the mouth piece firmly with the lips	( )	( )
6	Tilt your head back slightly	( )	( )
7	Press the inhaler while starting a slow, deep breath	( )	( )
8	Continue to breathe in slowly and deeply for 5 seconds	( )	( )
9	Remove the inhaler from mouth and hold breath for at least 10 seconds or as long as comfortable.	( )	( )
	Breathes out slowly	( )	( )
10	If more than one puff is required, wait for at least 1 min and repeat steps 3-9	( )	( )
11	After use, replace the mouth piece cover	( )	( )

12. Do your inhalation technique re-checked by health care workers? a. Yes b. No
13. How frequently do they re-check? First visit/every visit

Observation check-list on inhalation of Dry Powder Inhaler (DPI) Steps

S. N	Steps	Performed	Not Performed
1	Insert the capsule into the hole of inhaler device as advised	( )	( )
2	Hold the top of the inhaler device firmly with one hand. Rotate the base until the capsule breaks	( )	( )
3	Breathe out fully, away from the inhaler. Place the inhaler device between lips as directed.	( )	( )
4	Breathe in strongly for as long as you can to get powder into airway	( )	( )
5	Remove the inhaler device from the mouth	( )	( )
6	Hold your breath for 10 seconds as long as you find comfortable	( )	( )
7	Breathe out slowly, away from the inhaler	( )	( )
8	Make sure the capsule is empty. Repeat if medicine (powder) is left after 5 minutes	( )	( )
9	Rinse your mouth with water after you use inhaler, as directed by your physician. Do not swallow the rinse water	( )	( )
10	Close the mouthpiece or replace the cap after each use.	( )	( )
11	Store the inhaler in a cool, dry place	( )	( )
12	Clean the inhaler at least once a week with a dry cloth, as directed	( )	( )

13. Do your inhalation technique re-checked by health care workers? a. Yes b. No
14. How frequently do they re-check? First visit/every visit

Part 5

COPD Self-Management Scale

S. No.	Domains	Never	Rarely	Sometimes	Often	Always
	**Symptom Management**					
1	I practice breathing exercises when I do not feel shortness of breath.					
2	I inhale bronchodilators when I feel shortness of breath.					
3	I perform energy saving strategies (rest) when I feel shortness of breath.					
4	I seek help (e.g. call an ambulance or relatives) when the shortness of breath worsens rapidly.					
5	I automatically follow the doctor's prescription instructions.					
6	I do not take anti-inflammatory drugs when I feel serious shortness of breath.					
	**Daily Life Management**					
7	I exercise more than 2-3hours per week when my disease is stable					
8	I adjust my exercise intensity according to my health status (e.g. just walk around the house when I feel uncomfortable).					
9	I try to avoid inhaling dust, smoke and noxious fumes					
10	I avoid eating foods containing too much sugar or calories					
11	I limit eating sodium containing foods					
12	I avoid gas forming or bloating foods					
13	I used to take 4-6 small meal per day instead of taking large meals					
14	I used to check my weight regularly					
	**Emotion Management**					
15	I often encourage myself to be optimistic					
16	I talk with relatives and friends when I feel upset or anxious.					
17	I seek help and consolation with my family or friends when I feel down					
18	I try my best to change the thought that					

	I am a burden to the family.					
19	I exchange psychological experiences with other patients with similar diseases.					
	**Information Management**					
20	I discuss with medical staff about issues related to the disease.					
21	I keep complete records of my disease related documents (e.g. patient medical records, medical examination results).					
22	I make a list of all my questions when I consult with medical staff.					
	**Self-efficacy**					
23	I can actively stick to the doctor's prescription instructions.					

Emotional Distress

GAD-7 Anxiety

**B. Over the last 2 weeks, how often have you been bothered by any of the following problems?**		**Not at all (0)**	**Several days (1)**	**More than half the days (2)**	**Nearly everyday (3)**
1	Feeling nervous anxiety or on edge				
2	Not being able to stop or control worrying				
3	Worrying too much about different things				
4	Trouble relaxing				
5	Being so restless that it is hard to sit still				
6	Becoming easily annoyed or irritable				
7	Feeling afraid as if something awful might happen				

Column total of GAD-7 Score ☐ = _____ + _____ + _____

Note: Scores of 5, 10, and 15 represent cut points for mild, moderate, and severe anxiety, respectively.

PHQ-9, Depression

D. Over the last 2 weeks, how often have you been bothered by any of the following problems?		Not at all (0)	Several days (1)	More than half the days (2)	Nearly everyday (3)
1	Little interest or pleasure in doing things				
2	Feeling down, depressed, or hopeless				
3	Trouble falling or staying asleep, or sleeping too much				
4	Feeling tired or having little energy				
5	Poor appetite or overeating				
6	Feeling bad about yourself — or that you are a failure or have let yourself or your family down				
7	Trouble concentrating on things, such as reading the newspaper or watching television				
8	Moving or speaking so slowly that other people could have noticed? Or the opposite – being so fidgety or restless that you have been moving around a lot more than usual				
9	Thoughts that you would be better off dead of or hurting yourself in some way				

PHQ-9 Score = _____ + _____ + _____

Note: Scores of 5, 10, 15, and 20 represent cut points for mild, moderate, moderately severe and severe depression, respectively.

Thank You very Much
